# Ultrashort broadband polarization beam splitter based on a combined hybrid plasmonic waveguide

**DOI:** 10.1038/srep19609

**Published:** 2016-01-20

**Authors:** Ken-Wei Chang, Chia-Chien Huang

**Affiliations:** 1 Department of Physics, National Chung Hsing University, 250, Kuo Kuang Rd. Taichung, 402, Taiwan, R.O.C; 2 Institute of Nanoscience, National Chung Hsing University, 250, Kuo Kuang Rd. Taichung, 402, Taiwan, R.O.C

## Abstract

We propose an ultracompact broadband polarization beam splitter (PBS) based on a combined hybrid plasmonic waveguide (HPW). The proposed PBS separates transverse-electric (TE) and transverse-magnetic (TM) modes using a bent lower HPW with vertical nanoscale gaps and a straight upper HPW with a horizontal nanoscale gap, respectively, without relying on an additional coupling region. This design considerably reduces the length of the PBS to the submicron scale (920 nm, the shortest PBS reported to date) while offering polarization extinction ratios (PERs) of ~19 dB (~18 dB) and insertion losses (ILs) of ~0.6 dB (~0.3 dB) for the TE (TM) mode over an extremely broad band of 400 nm (from *λ* = 1300 nm to 1700 nm, covering entirely second and third telecom windows). The length of the designed PBS can be reduced further to 620 nm while still offering PERs of 15 dB, realizing a densely photonic integrated circuit. Considering the fabrication tolerance, the designed PBS allows for large geometrical deviations of ±20 nm while restricting PER variations to within 1 dB, except for those in the nanoscale gaps smaller than 10nm. Additionally, we also address the input and ouput coupling efficiencies of the proposed PBS.

To fulfill ever-increasing transmission demands of optical communication systems, polarization division multiplexing (PDM) plays a pivotal role in manipulating optical signals for chip-scale photonic integrated circuits (PICs)^1^,^2^,^3^,^4^,^5^. Polarization beam splitters (PBSs), which separate transverse-electric (TE) and transverse-magnetic (TM) modes, are essential components for PDM^4^ and allow the two polarization modes to be processed independently, doubling the traffic bandwidth. Numerous criteria used to assess PBSs include device dimensions, polarization extinction ratios (PERs), insertion losses (ILs), operating bandwidths, fabrication tolerances, and structure complexities. Among these, minimizing PBS dimensions while retaining satisfactory device performance is desirable for constructing coherent receivers and is of vital importance for developing next-generation ultradense PICs. Over the years, many types of PBSs^5^,^6^,^7^,^8^,^9^,^10^,^11^,^12^,^13^,^14^,^15^,^16^,^17^,^18^,^19^,^20^,^21^,^22^,^23^,^24^,^25^,^26^,^27^,^28^,^29^,^30^,^31^,^32^,^33^,^34^,^35^ have been reported that have utilized various designs and have included adiabatic mode evolution (AME) devices^6^,^7^, directional couplers (DC)^8^,^9^,^10^,^11^,^12^,^13^,^14^,^15^,^16^,^17^,^18^,^19^,^20^, multimode interference (MMI) devices^21^,^22^,^23^,^24^,^25^, Mach–Zehnder interferometers (MZI)^26^,^27^,^28^, photonic crystals (PhC)^29^,^30^,^31^, and grating^31^,^32^,^33^ structures. Most PBSs^6^,^7^,^8^,^9^,^10^,^11^,^12^,^13^,^14^,^15^,^16^,^17^,^18^,^19^,^20^,^21^,^22^,^23^,^24^,^28^,^32^,^33^,^34^ have adopted silicon-on-insulator (SOI) platforms to effectively decrease device dimensions by utilizing the high-index contrast properties of these platforms.

To achieve satisfactory PERs, AME-based PBSs^6^,^7^ must be very long (>200 μm) because of their slowly evolving geometries, but they have less stringent fabrication tolerance and broadband operation requirements. Although the device lengths of DC-based PBSs^8^,^9^,^10^,^11^,^12^,^13^,^14^,^15^,^16^,^17^,^18^,^19^,^20^ can be reduced to several to tens of micrometers with reasonable PERs (10–20 dB), operating bandwidths are narrower than those of AME-based PBSs because of the requirement of utilizing phase-matched modes with a precisely tuned coupling. MMI-based PBSs^21^,^22^,^23^,^24^,^25^ have a simpler fabrication process and larger fabrication tolerance than those of AME-based PBSs; however, dimensions of conventional MMI devices^35^,^36^ are determined by the common multiple of the self-imaging lengths^37^ of TE and TM modes, resulting in very long devices (>1000 μm). To shorten the lengths of MMI-based PBSs, some innovative designs have recently been reported, including two-mode interference^21^ (~8.8 μm), 2 × 2 two-mode interference^22^ (~0.94 μm for the length of MMI section only other than the whole PBS, the lengths for the input/output part should be included), metal–insulator–metal (MIM)-embedded^22^ (~44 μm), hybrid plasmonic waveguide (HPW)^24^ (~2.5 μm), and cascaded^25^ (<950 μm) MMIs. To date, the shortest PBS reported was obtained for an MMI that utilized hybrid plasmonic waveguide (HPW)^24^ and achieved submicron length with a PER >10 dB over a 80 nm bandwidth. MZI-based PBSs^26^,^27^,^28^, in addition to requiring highly birefringent materials, have had device lengths that were too long (300–3000 μm). Other options that can yield device lengths of tens of micrometers are devices that use the PhC-based PBSs^29^,^30^,^31^ and grating-based PBSs^32^,^33^,^34^. The disadvantages of the former are fabrication complexity and relatively large loss due to the scattering; the latter, besides having a similarly complicated fabrication process, are also difficult to integrate into PICs.

Among the aforementioned PBSs^6^,^7^,^8^,^9^,^10^,^11^,^12^,^13^,^14^,^15^,^16^,^17^,^18^,^19^,^20^,^21^,^22^,^23^,^24^,^25^,^26^,^27^,^28^,^29^,^30^,^31^,^32^,^33^,^34^,^35^,^36^, DC-based PBSs^8^,^9^,^10^,^11^,^12^,^13^,^14^,^15^,^16^,^17^,^18^,^19^,^20^ have become the most popular, due to their structural simplicity, satisfactory performance, and diverse designs. In a DC-based PBS, one selected mode is separated using evanescent field coupling to the cross bar, while the remaining mode propagates straight along the through bar. Theoretically, selecting highly polarization-dependent materials is beneficial for improving the PERs and shortening the coupling length, and therefore, a high-index-contrast SOI platform^8^,^9^,^11^,^15^,^17^,^20^ is often utilized. In addition to using high-index-contrast dielectrics, metals exhibit stronger birefringences induced by exciting the surface plasmon polariton (SPP) guided modes^38^, for which the majority of the electric field must be perpendicular to the metal surface. Moreover, the confinement of SPP modes that break the diffraction limit^38^ is also advantageous as it significantly improves the degree of integration of photonic devices^39^,^40^,^41^,^42^. However, the inherent ohmic losses of SPP modes are much larger than those of dielectric-guided modes.

Considering the trade-off between mode confinement and propagation loss, an HPW structure^43^,^44^,^45^,^46^,^47^ consisting of a low-index spacing layer between a high-index dielectric and a metal has been proposed to significantly reduce the ohmic loss by forming a hybrid plasmonic mode by coupling a pure SPP mode and a dielectric waveguide mode. As a result, many DC-based PBSs using HPWs^11^,^12^,^13^,^14^,^16^,^18^,^19^ have been reported recently to further reduce the dimensions of PBSs while having reasonable propagation lengths of tens of micrometers. A short PBS^11^ of 1.1 μm was proposed using nanoscale silver cylinders to perform polarization selection between two silicon waveguides. The PERs were 22.1 dB and 23.1 dB for TE and TM modes, respectively. However, the numerical calculations were limited to a two-dimensional structure. Guan *et al*.^12^ reported an asymmetrical directional coupler consisting of an HPW and a silicon nanowire. The length of the PBS was 3.7 μm, and the PERs of the two polarized modes were about 12 dB. In refs 13 and 14, both of the PBSs were also based on asymmetrical DC structures. In ref. 13, the asymmetrical DC consisted of a horizontally slotted waveguide and an HPW. The length of the PBS was 5 μm with PERs of about 20 dB. In ref. 14, the PBS consisted of a strip dielectric waveguide and an HPW. The device length was 4.13 μm and the PERs were 16.4 dB and 20.9 dB for TE and TM modes, respectively. Another design using a three-port DC^16^,^18^ was reported to achieve higher PERs (>20 dB). However, the device lengths were longer than those^13^,^14^ of asymmetrical DC structures. The shortest three-dimensional DC-based PBS with an HPW structure^19^ (~2.5 μm) adopted a copper nanorod array placed between two silicon waveguides. Using the localized surface plasmon resonance between the silicon waveguides, the TE mode was effectively coupled to the cross-channel, significantly reducing the device length and yielding a PER of ~15 dB.

In this paper, we propose an innovative design for a PBS based on a combined HPW (CHPW) consisting of two parts: a bent lower HPW deposited on a SOI platform and formed by a high-index silicon (Si) core sandwiched between two low-index nanoscale layers of silicon dioxide (SiO_2_) and Ag and a straight upper HPW formed by a nanoscale layer of SiO_2_ sandwiched between Si and Ag layers. In this way, TE and TM modes are supported by the bent lower and straight upper HPWs, respectively, without the requirement of a coupling region that is typically indispensable in DC-based PBSs. The key idea allows the length of the proposed PBS to be shortened to the submicron scale while retaining satisfactory PERs and broad operating bandwidths. Furthermore, the fabrication tolerance is also discussed in detail to assess the feasibility of the designed PBS.

## Results

### Design and mode properties of the proposed PBS

**Figure 1:**
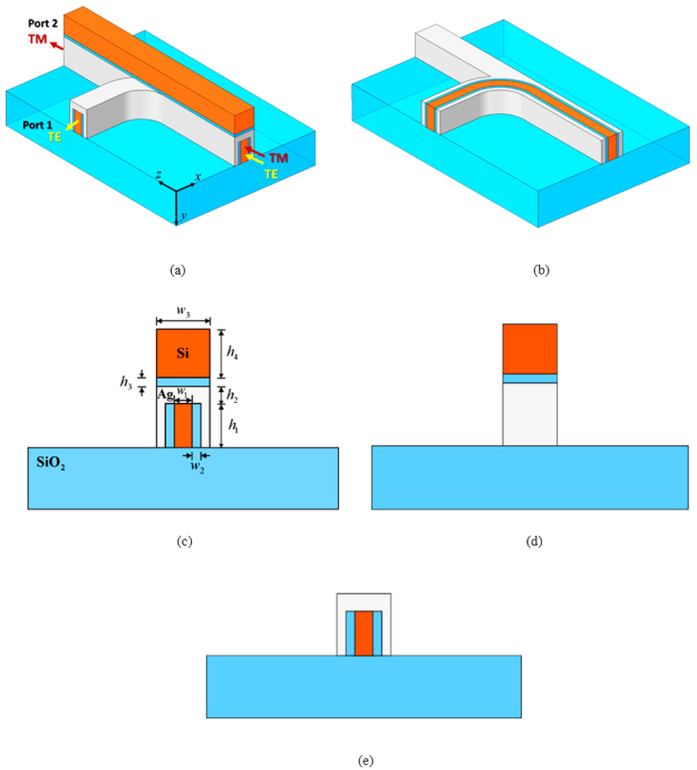
(**a**) 3D schematic of proposed PBS, which is surrounded by air (white region). Upper part is formed by SiO_2_ (depicted in blue) sandwiched between Si (depicted in orange) and Ag (depicted in gray) and guides the TM mode. Lower part is formed by Si that is surrounded by SiO_2_ and covered by Ag and guides the TE mode. (**b**) Proposed PBS lifted off from upper Si, SiO_2_, and some of Ag, leaving interior structure of lower part clearly visible. Cross-sections of (**c**) input port and (**d**) output port for transmitting TM mode (*i*.*e*., majority of electric field is in *y* direction), and (**e**) output port for transmitting TE mode (*i*.*e*., majority of electric field is in *x* direction).

The schematic of the proposed PBS (Fig. 1(a)) consists of two HPW structures deposited on a SiO_2_ substrate (depicted in blue). To clearly view the interior structure, Fig. 1(b) shows the bent lower HPW structure of the proposed PBS lifted off from the upper Si (depicted in orange), SiO_2_, and Ag (depicted in gray) layers. The bent lower HPW structure is formed by a Si core that is sandwiched successively between SiO_2_ and Ag layers, and the straight upper HPW structure (Fig. 1(a)) that is stacked on the lower part is formed by a horizontal SiO_2_ layer sandwiched between Si and Ag. According to the HPW mechanism, TE (*i*.*e*., the majority of the electric field is in the x direction) and TM (*i*.*e*., the majority of the electric field is in the y direction) modes are guided through the lower and upper parts, respectively, and the energies of the two modes are concentrated primarily in the SiO_2_ nanoscale layers. The cross-sectional views of the proposed PBS at the input port, at the output port for transmitting the TM mode (port 2), and at the output port for transmitting the TE mode (port 1) are shown in Fig. 1(c) (with the geometrical parameters), 1(d), and 1(e), respectively. Note that the novel design of the proposed PBS allows TE and TM modes to be separated by the bent lower and straight upper HPW structures, respectively, without the requirement of a coupling region, making the length of the designed PBS extremely short. Here, the 90° bent waveguide helps to decouple the two modes very clearly and thus improves the performance of the proposed PBS.

**Figure 2:**
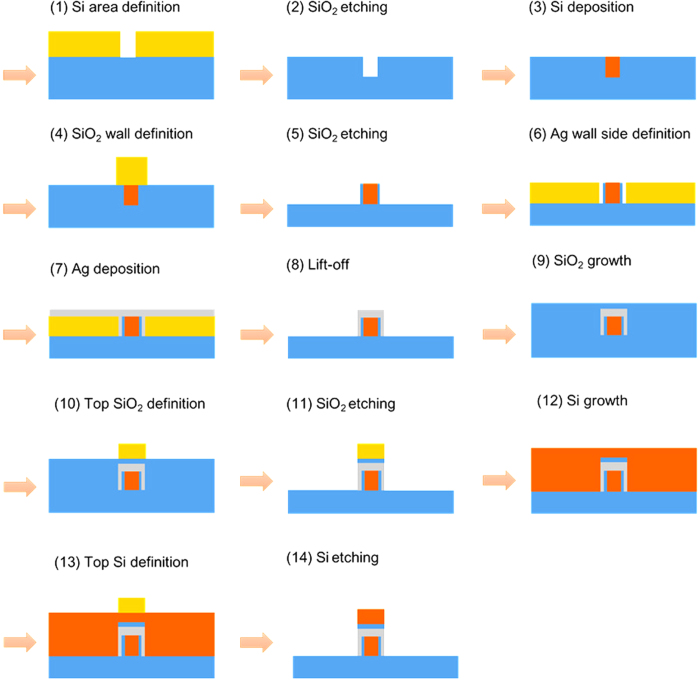
Schematic diagram of the fabrication processes for the proposed PBS.

The fabrication processes of the proposed device are schematically shown in Fig. 2. First of all, the patterned hard masks of the bent TE and straight TM channels are fabricated by using high resolution electron beam lithography (EBL). After that, we proceed to carry out the following steps. (1) A SiO_2_ substrate (blue) is prepared for depositing with a negative photoresist (PR) thin film (yellow) to define the inner Si region by the preceding mask of the bent TE channel, a PR exposure with ultraviolet (UV) light, development, and an etching process. (2) A well with width *w*_1_ and height *h*_1_ is formed by etching SiO_2_ and lifting off the PR film. (3) A Si layer with height of *h*_1_ is deposited using chemical vapor deposition (CVD) in the well. After that, we proceed chemical mechanical polishing (CMP) process to obtain a flat plane. (4) The SiO_2_ walls at both sides of Si with width of 2*w*_2_ + *w*_1_ are defined by the processes including a PR film deposition, a patterned mask, a PR exposure, development, and an etching process. (5) With the help of a patterned mask, two SiO_2_ vertical walls are formed by reactive ion etching (RIE) and then the PR is lifted off. (6) The Ag regions with width of *w*_3_ − 2*w*_2_ − *w*_1_ are first defined by using the patterned mask of bent TE channel and depositing PR film. Next, the TM channel is defined by lifting off the PR film of that part using the patterned mask of TM channel. (7) An Ag layer with height of *h*_1_ + *h*_2_ is deposited. (8) Lifting off the PR film forms the final bent TE channel and the straight TM channel without SiO_2_ and Si layers yet. (9) A SiO_2_ layer with height of *h*_1_ + *h*_2_ + *h*_3_ is deposited using thermal oxidation. Next, using CMP to obtain a flat SiO_2_ surface. (10) After depositing PR film, we use the patterned mask of TM channel, a PR exposure, development, and an etching process to form PR film with width of *w*_3_. (11) With the help of a patterned mask, etching SiO_2_ by RIE forms the SiO_2_ layer with width of *w*_3_ and height of *h*_3_ on the top of Ag layer of the straight TM channel. (12) After removing the PR film, a Si layer is deposited with height of *h*_1_ + *h*_2_ + *h*_3_ + *h*_4_. Next, using CMP to obtain a flat Si surface. (13) After depositing PR film on the Si layer, using the patterned mask of TM channel, a PR exposure, and development to form PR film with width of *w*_3_. (14) Finally, the proposed device is formed by etching Si and removing the PR film.

**Figure 3:**
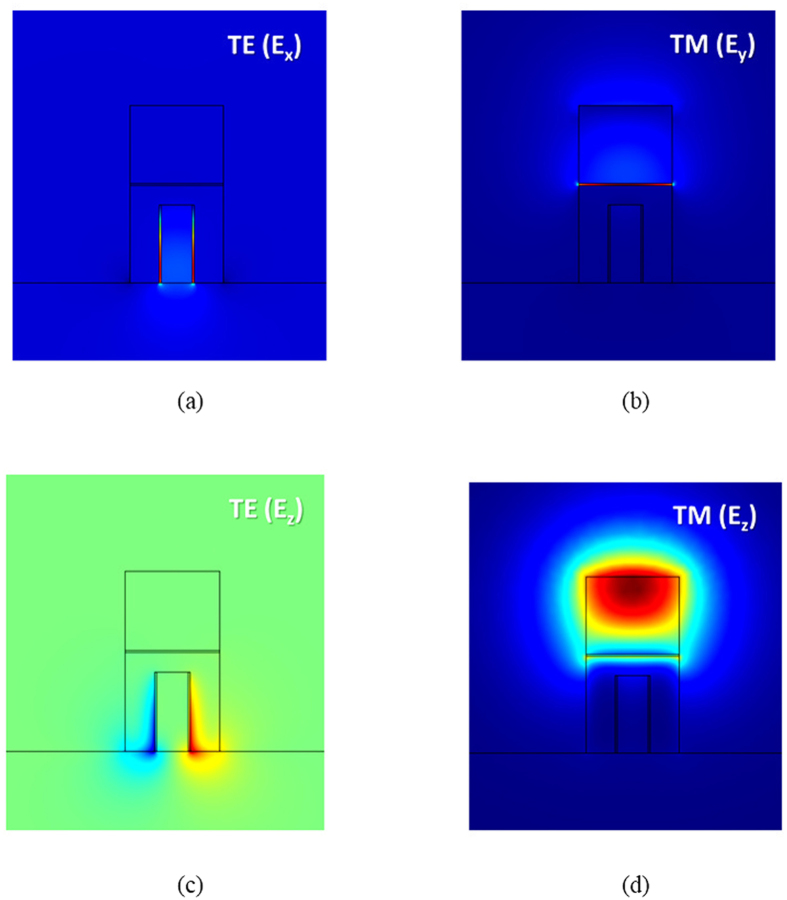
(**a**) Electric field profile, *E*_*x*_, of TE, (**b**) electric field profile, *E*_*y*_, of TM, (**c**) electric field profiles, *E*_*z*_, of TE, and (**b**) electric field profiles, *E*_*z*_, of TM modes under the conditions *w*_1_ = 80 nm, *w*_2_ = 5 nm, *w*_3_ = 240 nm, *h*_1_ = 200 nm, *h*_2_ = 50 nm, *h*_3_ = 5 nm, and *h*_4_ = 200 nm.

To design an optimal PBS, we first analysed the mode properties of the proposed PBS. The relative permittivities of Si, SiO_2_, and Ag used in this model are *ε*_Si_ = 11.937^48^, *ε*_SiO2_ = 2.088^48^, and *ε*_Ag_ = −129.2 + 3.285*i*^49^, respectively, assuming operation at a telecommunication wavelength of *λ* = 1,550 nm. Considering the trade-off^50^ between the mode confinement and propagation lengths of the HPW modes, the selected geometrical parameters are *w*_1_ = 80 nm, *w*_2_ = 5 nm, *w*_3_ = 240 nm, *h*_1_ = 200 nm, *h*_2_ = 50 nm, *h*_3_ = 5 nm, and *h*_4_ = 200 nm (see Fig. 1(c)). The effective refractive indices of TE and TM modes were calculated to be 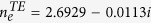 and 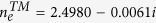, respectively (see methods). We observe that the major field profiles of TE (*E*_*x*_) and TM (*E*_*y*_) modes (as shown in Fig. 3(a,b), respectively) are mainly confined to the nanoscale SiO_2_ gaps because of the mode coupling effects of the dielectric waveguide and SPP mode in HPWs. Smaller SiO_2_ gaps yield increased energy confinement. To consider the fabrication effort, mode size, and device performance, we selected the width of the SiO_2_ gaps to be 5 nm, which is achievable with the present fabrication technology. In addition, the space of *h*_2_ = 50 nm is sufficient to effectively avoid evanescent field coupling between TE and TM modes. To quantitatively evaluate the mode properties of an HPW, the normalized mode area and propagation length were calculated (see methods). The results calculated for the present structure are *A*_*e*_/*A*_*o*_ = 3.1 × 10^−3^ (1.24 × 10^−2^) and *L*_*m*_ = 10.91 (20.18) μm for the TE (TM) mode. For the TE mode, the mode size is smaller than that of the TM mode because of the complete coverage by metal; however, one disadvantage is the higher ohmic losses of the metals. Figure 3(c,d) show the minor electric fields, *E*_*z*_, of TE and TM modes, respectively, which are responsible for metal attenuation^51^. Note that the magnitudes of the minor *E*_*z*_ fields shown in Fig. 3(c,d) are two orders of magnitudes smaller than those of the dominant *E*_*x*_ or *E*_*y*_ fields shown in Fig. 3(a,b). We observe that the *E*_*z*_ fields of TE and TM modes are concentrated in the metal and in the upper Si, respectively. Therefore, the ohmic loss of the TE mode (*L*_*m*_ = 10.91 μm) is larger than that of the TM mode (*L*_*m*_ = 20.18 μm), demonstrating the calculated propagation lengths. The numerical results above show that the proposed HPW structure is capable of accomplishing nanoscale field localization while offering sufficiently long propagation distances (tens of micrometers) and can thus be applied to construct an ultra-small PBS.

### Propagating performance of the proposed PBS

**Figure 4:**
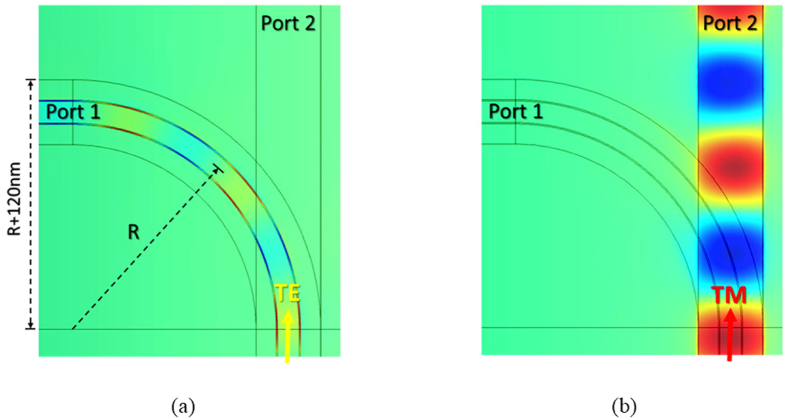
Field distributions of (a) TE and (**b**) TM modes along bent lower and straight upper HPWs of proposed PBS, respectively, at bending radius of *R* = 800 nm. Other geometric parameters are identical to those in Fig. 3.

**Figure 5:**
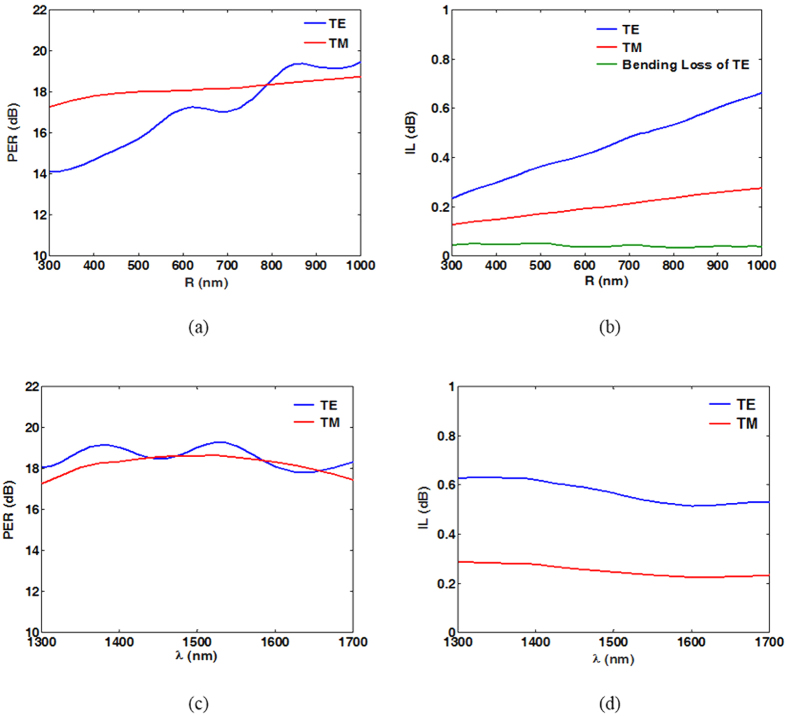
(**a**) Polarization extinction ratios (PERs) and (**b**) insertion losses (ILs) of TE and TM modes with the bending loss of the TE mode as functions of radius of curvature *R*. (**c**) Polarization extinction ratio (PER) and (**d**) insertion loss (IL) versus operating wavelength, λ.

**Figure 6:**
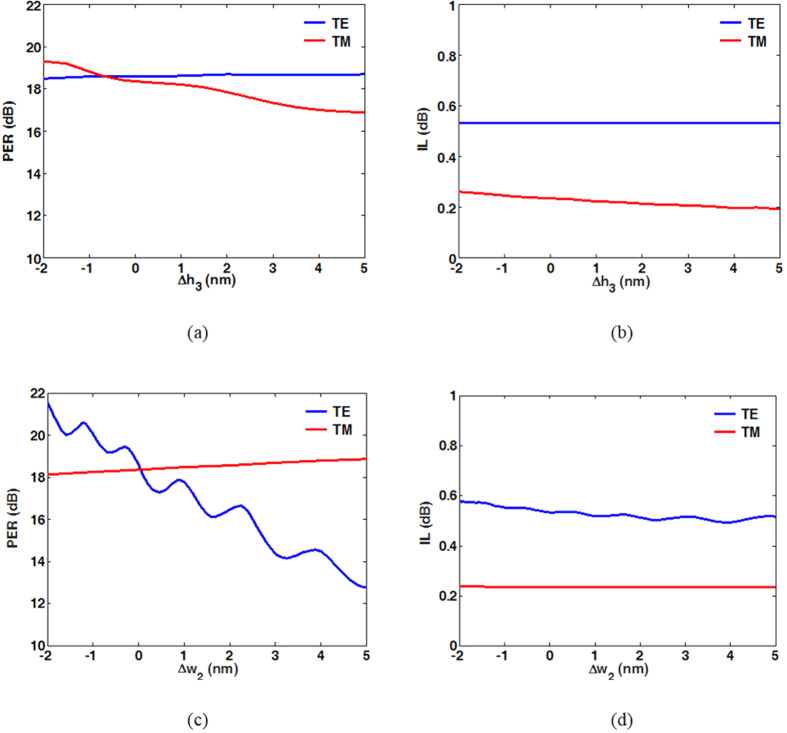
(**a**) Polarization extinction ratio (PER) and (**b**) insertion loss (IL) of proposed PBS versus variation of *h*_3_ (Δ*h*_3_),(**c**) Polarization extinction ratio (PER) and (**d**) insertion loss (IL) of proposed PBS versus variation of *w*_2_ (Δ*w*_2_).

The propagating field distributions of TE and TM modes are shown in Fig. 4(a,b), respectively, for a bending radius of *R* = 800 nm. We observe that the two modes are separated independently into the upper and lower parts of the proposed PBS. Unlike in typical DC-based PBSs^8^,^9^,^10^,^11^,^12^,^13^,^14^,^15^,^16^,^17^,^18^,^19^,^20^, no coupling region is required to split a specific mode from the input port, making the proposed PBS extremely short. To further analyse the device performance of the proposed PBS, the calculated PERs and ILs of the two modes as functions of *R* are shown in Fig. 5(a,b), respectively (see methods). In Fig. 5, PER_TM_ (~18 dB) and IL_TM_ (~0.2 dB) vary moderately as *R* increases because the TM mode propagates in the straight upper waveguide rather than in the bent lower one. In contrast, PER_TE_ and IL_TE_ increase significantly as *R* increases, as shown in Fig. 5. The increase in PER_TE_ as *R* increases results from the decreased bending radiation transferring to port 2. For the IL_TE_, the increase in propagation distance due to the increase in *R* leads to greater ohmic loss, as shown in Fig. 5(b). Additionally, bending loss of the TE mode as the variation of *R* is also shown in Fig. 5(b). Theoretically, IL_TE_ (total power loss) is the sum of the propagation and bending losses. We see that the variation of the bending loss is between 0.03 to 0.05dB without exhibiting monotonous decrease as the *R* increases because of the presence of another waveguide transmitting TM mode. As shown in Fig. 5(b), the major part of IL_TE_ is resulted from the propagation loss because the most power is highly confined in the vertical SiO_2_ layers surrounding by Ag. In particular, PER_TE_ and PER_TM_ exceed 15 dB and 18 dB, respectively, even though *R* is reduced to 500 nm. Under the condition *R* = 500 nm, the dimensions of the designed PBS are only 620 nm (length) × 620 nm (width) × 455 nm (height). Under the condition *R* = 800 nm, PER_TE_ further increases to above 18 dB. For the IL, which is responsible for the intrinsic losses of plasmonic waveguides, the increase of IL_TE_ as *R* increases is greater than that of IL_TM_, because the propagation loss of the TE mode (L_m_ = 10.91 μm) is approximately twice that of the TM mode (L_m_ = 20.18 μm). However, IL_TE_ (~0.6 dB at *R* = 800 nm) is still low. Overall, to achieve satisfactory performance (PER > 18 dB and IL < 0.6 dB at *R* = 800 nm), the dimensions of the proposed PBS must be about 920 nm × 920 nm × 455 nm (the smallest PBS yet designed), and thus, the proposed design has great potential to realize high-density PICs with good performance. Another pivotal characteristic for assessing a PBS is its operating bandwidth with satisfactory PER and IL. Considering the used material dispersions^48^,^49^, Fig. 5(c,d) show PER and IL versus the operating wavelength λ between 1,300 nm and 1,700 nm. The results show that the proposed PBS can be operated over a broad bandwidth of 400 nm with PER > 17 dB and IL < 0.6 dB for both modes. With operation over a narrower bandwidth of 200 nm (from 1400 nm to 1600 nm), the PERs can be improved to greater than 18 dB. From Fig. 5(c,d), we observe that the PERs and ILs of the designed PBS are wavelength-insensitive, because of the lack of phase-matched conditions with precise coupling that are required in DC- and MMI-based PBSs. Finally, the fabrication tolerance was also investigated to identify the geometric parameters that significantly affect the performance of the present design. The degradations of the PERs and ILs with variations in *w*_1_, *w*_3_, *h*_1_, *h*_2_, and *h*_4_ were all within 1 dB and 0.2 dB, respectively, even when these parameters were varied by up to ±20 nm. This stability results from the majority of the energies of the hybrid SPP modes being concentrated in the thin SiO_2_ layers (*i.e*., those with the geometric parameters of *h*_3_ and *w*_2_). Consequently, we first studied the PERs and ILs while varying *h*_3_ (Δ*h*_3_), and the results are shown in Fig. 6(a,b). The considered values of Δ*h*_3_ range from −2 nm to 5 nm, because the originally designed thickness *h*_3_ was only 5 nm. For the TM mode, the values of PER_TM_ and IL_TM_ were moderately influenced by Δ*h*_3_. It can be understood that a large portion of the energy resides in the upper Si region, in addition to that concentrated in the thin SiO_2_ gap. In contrast, as expected, PER_TE_ and IL_TE_ are approximately constant as *h*_3_ varies. The other critical geometrical parameter is the width (*w*_2_) of the vertical SiO_2_ regions supporting the TE mode. The calculated PERs and ILs are shown in Fig. 6(c,d), respectively. Clearly, PER_TM_ and IL_TM_ are slightly influenced by Δ*w*_2_, as expected. In contrast to PER_TM_, PER_TE_ is significantly influenced by Δ*w*_2_, as shown in Fig. 6(c). This difference results from greater values of *w*_2_ causing looser energy confinement in the TE mode. Therefore, more energy is coupled to port 2 because of larger bending radiation. This causes PER_TE_ to be reduced significantly. In contrast, shrinking the width of SiO_2_ increases PER_TE_ due to better energy confinement. From the above discussions of the fabrication tolerances, the width *w*_2_ results in the most significant influence on PER_TE_. As a result, we conclude that the ability to precisely control the critical parameter *w*_2_ determines the PER_TE_ performance of the proposed PBS. Fortunately, the other geometrical parameters besides *w*_2_ have more moderate influences on the PERs and ILs. The results confirm the high fabrication tolerances of the proposed PBS, except for with respect to Δ*w*_2_. For further improving the PERs of TE and TM simultaneously to better values larger than 20 dB, we can decrease the thicknesses of SiO_2_ layers between Si and Ag. For instance, the PERs of the TE mode are 21.0 dB and 22.1 dB at the conditions of *w*_2_ = 4 nm and 3 nm, respectively, as shown in Fig. 6 (c). As for the TM mode, the PER is 20.2 dB at the condition of *h*_3_ = 2 nm, which is not involved in Fig. 6(a). Certainly, the fabrication precision will be severer. However, if only the higher PER_TE_ is concerned, increasing the radius of curvature of the bent waveguide to 1100 nm can reach 20.85 dB.

### The coupling efficiencies of the proposed PBS

**Figure 7:**
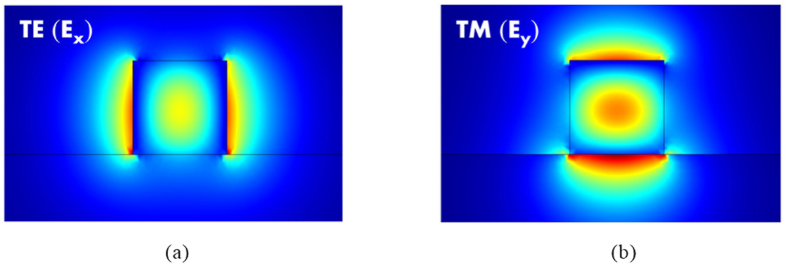
Electric field distributions of (a) TE and (**b**) TM modes of a Si stripe waveguide with width of 300 nm and height of 300 nm. The two modes are provided as the input beams coupling into the proposed PBS.

**Figure 8:**
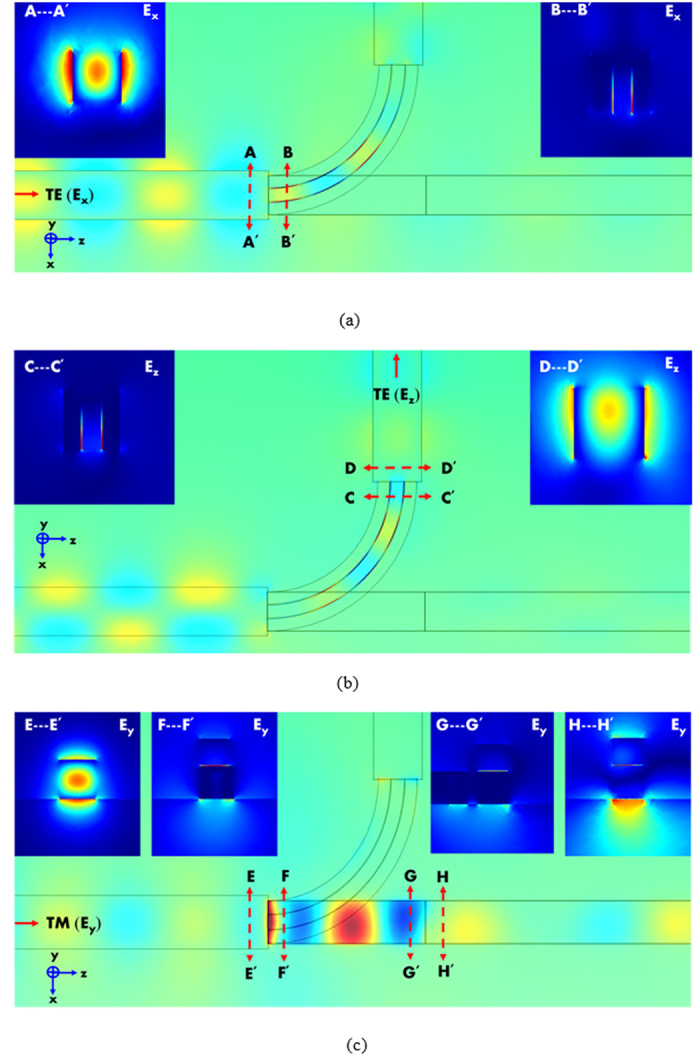
(**a**) The propagating field distribution of *E*_*x*_ of the TE mode for the condition of *R* = 800nm. (**b**) The propagating field distribution of *E*_*z*_ of the TE mode. The insets show the field distributions 100 nm before (CC′) and after (DD′) the output coupling interface. (**c**) The propagating field distribution of *E*_*y*_ of the TM mode. The insets show the field distributions 100 nm before (EE′) and after (FF′) the input coupling interface, and that 100 nm before (GG′) and after (HH′) the output coupling interface.

Considering the practical applications of the proposed PBS, we address the input and output coupling efficiencies in detail. The light is coupled into the input port by a Si stripe waveguide with a width of 300 nm and a height of 300 nm supporting both the fundamental TE and TM modes at *λ* = 1.55 μm. The output port for transmitting TE mode is connected with the same Si stripe waveguide as the input one. Nevertheless, the output port for transmitting TM mode is connected with a slot^52^ waveguide with the same size and materials as those of the TM channel but replacing the lower Ag with Si. This is because a slot waveguide facilitates to achieve a higher coupling efficiency than that of a Si stripe waveguide. The input sources of the TE and TM modes calculated by the boundary mode solver are shown in Fig. 7(a,b), respectively. Once launching the TE mode of the Si stripe waveguide into the proposed PBS, we obtain the propagating field distribution (*E*_*x*_) for the condition of *R* = 800nm as shown in Fig. 8(a). The field distributions 100nm before (indicated by a dashed red line between AA′) and after (indicated by a dashed red line between BB′) the input coupling interface are also shown in the insets of Fig. 8(a). Here, the input coupling efficiency is determined by the ratio of powers along the z-direction at the planes AA′ and BB′, and the calculated value is ~94.5%. In addition, the propagating field distribution (*E*_*z*_) of the TE mode is also shown in Fig. 8(b) for clearly displaying the output field distribution because the TE mode power flows along a bent waveguide from the *z*-direction (input coupling) to the minus *x*-direction (output coupling). Likewise, the field distributions 100 nm before (CC′) and after (DD′) the output coupling interface are shown in the insets of Fig. 8(b). The definition of the output coupling efficiency is the ratio of powers flowing along the minus *x*-direction at the planes CC′ and DD′, and the calculated value is ~94.7%. Note that the input and output coupling efficiencies are almost identical due to the same structure connected. For completely analyzing the performances of the proposed PBS, we calculate the total coupling efficiency of the TE mode defined by the ratio of power between the planes AA′ (the power flows along the *z*-direction) and DD′ (the power flows along the minus *x*-direction). The calculated value of the total coupling efficiency is ~58.5%. In Fig. 5(b), we observe that the IL_TE_ at *R* = 800nm is 0.533dB (power transmitted ratio within the bent waveguide is ~88.3%) and the bending loss is fairly low relative to the propagating loss of a highly confined plasmonic waveguide mode. Comparing with the total coupling efficiency of ~58.5%, we calculate the total power transmitting ratio (~79.1%) by summing up the input (~94.5%), output (~94.7%), bending, and propagating (~88.3%) losses. We found that the obvious difference of ~20.6% between the two calculations is resulted from the stronger bending loss of while coupling with the TE mode of the Si stripe waveguide (not highly confined mode) than that of the guided plasmonic TE mode (highly confined mode). Now, we turn attention to the TM mode analysis. The propagating field distribution (*E*_*y*_) of the TM mode and the field distributions at the planes of EE′, FF′, GG′, and HH′ are shown in Fig. 8(c). The input coupling efficiency of the TM mode is obtained by the ratio of the powers at the planes EE′ and FF′, and the value of FF′/EE′ is ~88.5%. Similarly, the output coupling efficiency of the TM mode is obtained by the ratio of the powers at the planes GG′ and HH′, and the value of HH′/GG′ is ~95.2%. For the TM mode, the total coupling efficiency is the ratio of the powers at the planes EE′ and HH′, and the calculated value is ~77.3%. Comparing with the total transmitting power ratio (~79.8%) of the TM mode, the two calculations have quiet close results as expected because the TM mode propagates in a straight path without bending loss.

We have reported a novel PBS based on a combined HPW. The proposed PBS consisted of two HPW structures arranged vertically. The bent lower HPW deposited on an SOI platform was formed by a silicon core sandwiched successively between silicon dioxide (SiO_2_) with nanoscale gaps and Ag, and the straight upper HPW was formed by a nanoscale gap of SiO_2_ sandwiched between Si and Ag layers. The innovative concept of the proposed PBS was to separate TE and TM modes with bent lower and straight upper HPWs, respectively, without coupling one of the guiding modes to the adjacent channel. As a result, the length of the designed PBS became extremely short. With dimensions of 920 nm (length) × 920 nm (width) × 455 nm (height), which make it the smallest PBS to date, the PER of the TE (TM) mode was ~19 dB (18 dB), and the IL was ~0.6 dB (0.3 dB) over an extremely broad band of 400 nm (from *λ* = 1300 nm to 1700 nm). In particular, the wavelength insensitivity of the proposed PBS properties resulted from avoiding the phase-matching requirement of DC-based PBSs. The input and output coupling efficiencies of the TE and TM modes are also addressed. For the TE mode, both the input and output ports are connected with Si stripe waveguides, and the two coupling efficiencies are about 94.5%. For the TM mode, the input coupling efficiency connected with a Si stripe waveguide is about 88.5% and the output efficiency connected with a slot waveguide is about 95.2%. Adding in the propagating ohmic loss and bending loss of the proposed PBS, the total coupling efficiencies of the TE and TM modes are about 58.5% and 77.3%, respectively. When the dimensions were further reduced to 620 nm × 620 nm × 455 nm, the proposed PBS still retained high PERs of >15 dB. These results indicate that the proposed PBS has the potential to realize high-density PICs with satisfactory performances.

## Methods

In this study, two kinds of properties including modal characteristics and transmission performances of the proposed PBS are calculated numerically.

### Design and mode properties of the proposed PBS

First, the modal properties at the input port are obtained by solving the Helmholtz equation using the boundary mode analysis of the simulation software COMSOL^TM^ Multiphysics, which is based on the finite element method (FEM). The normalized mode area (*A*_*e*_/*A*_*o*_) and propagation length (*L*_*m*_ = *λ*/[4πIm(*n*_*e*_)]) of a guided mode are essential features to characterize the figure of merit (FOM) of a plasmonic waveguide^44^, where *A*_*o*_ = *λ*^2^/4 denotes the diffraction-limited area of light in a vacuum and *λ* is the operating wavelength. The effective mode area, *A*_*e*_, in Eq. (1) denotes the ratio between the total mode energy, *W*_*m*_, and the peak value of the energy density, *W*(***r***), which is defined in Eq. (2):^44^


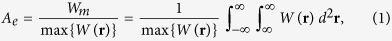


and


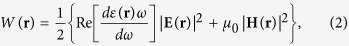


where *ω* is the angular frequency, *ε*(**r**) is the relative permittivity, *μ*_0_ is the vacuum permeability, and |**E**(**r**)|^2^ and |**H**(**r**)|^2^ are the intensities of the electric and magnetic fields, respectively. The propagation length represents the distance over which the mode energy intensity is attenuated to 1/*e* of the input light, where Im(*n*_*e*_) is the imaginary part of the effective mode index. The calculated window (500 × 5000 μm^2^) with the scattering boundary condition mimicking the necessary open boundary for solving mode characteristics is enough large to ensure no interference from boundary to affect the results. In addition, convergence is also tested by fining the meshes for certain thin regions and sharply variant fields.

### Propagating performance of the proposed PBS

After obtaining the mode characteristics of the designed PBS, we studied its performance by launching the TE and TM SPP modes into the input port. For evaluating the transmission characteristics of a PBS, we need to investigate the PERs and ILs of a specific mode. Here, the TE and TM modes are defined in Eqs (3) and (4), respectively:^11^,^12^,^19^


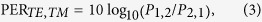


and


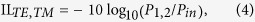


where *P*_*i*_ is the mode power at port *i* (*i* = 1, 2, or input).

## Additional Information

**How to cite this article**: Chang, K.-W. and Huang, C.-C. Ultrashort broadband polarization beam splitter based on a combined hybrid plasmonic waveguide. *Sci. Rep*. **6**, 19609; doi: 10.1038/srep19609 (2016).
